# Supplementary material for: An integrated workflow for quantitative analysis of the newly synthesized proteome
Source: Nat Commun. 2023 Dec 12;14:8237. doi: 10.1038/s41467-023-43919-3 (PMC10716174; doi:10.1038/s41467-023-43919-3)
Supplement: Supplementary file 1 — Supplementary Information [file 41467_2023_43919_MOESM1_ESM.pdf]

Supplementary Information for

An integrated workflow for quantitative analysis of the newly synthesized proteome

Toman Borteçen[1,2], Torsten Müller[3], and Jeroen Krijgsveld[1,3,*]

[1]German Cancer Research Center (DKFZ), Im Neuenheimer Feld 581, Heidelberg, Germany

[2]Heidelberg University, Faculty of Biosciences, Im Neuenheimer Feld 581, Heidelberg, Germany

[3]Heidelberg University, Medical Faculty, Im Neuenheimer Feld 581, Heidelberg, Germany

*Correspondence to J.K., email j.krijgsveld@dkfz.de, phone: +49-6221-421720

Supplementary Figures

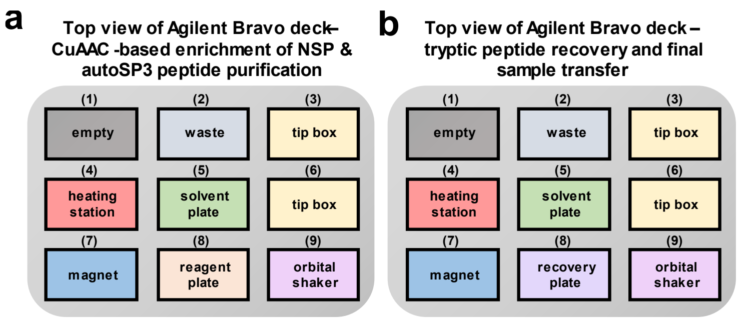

Supplementary Figure 1

Schematic overview of the Bravo liquid handling platform deck for the automated NSP enrichment and SP3 peptide purification (A) and recovery step of tryptic peptides (B).

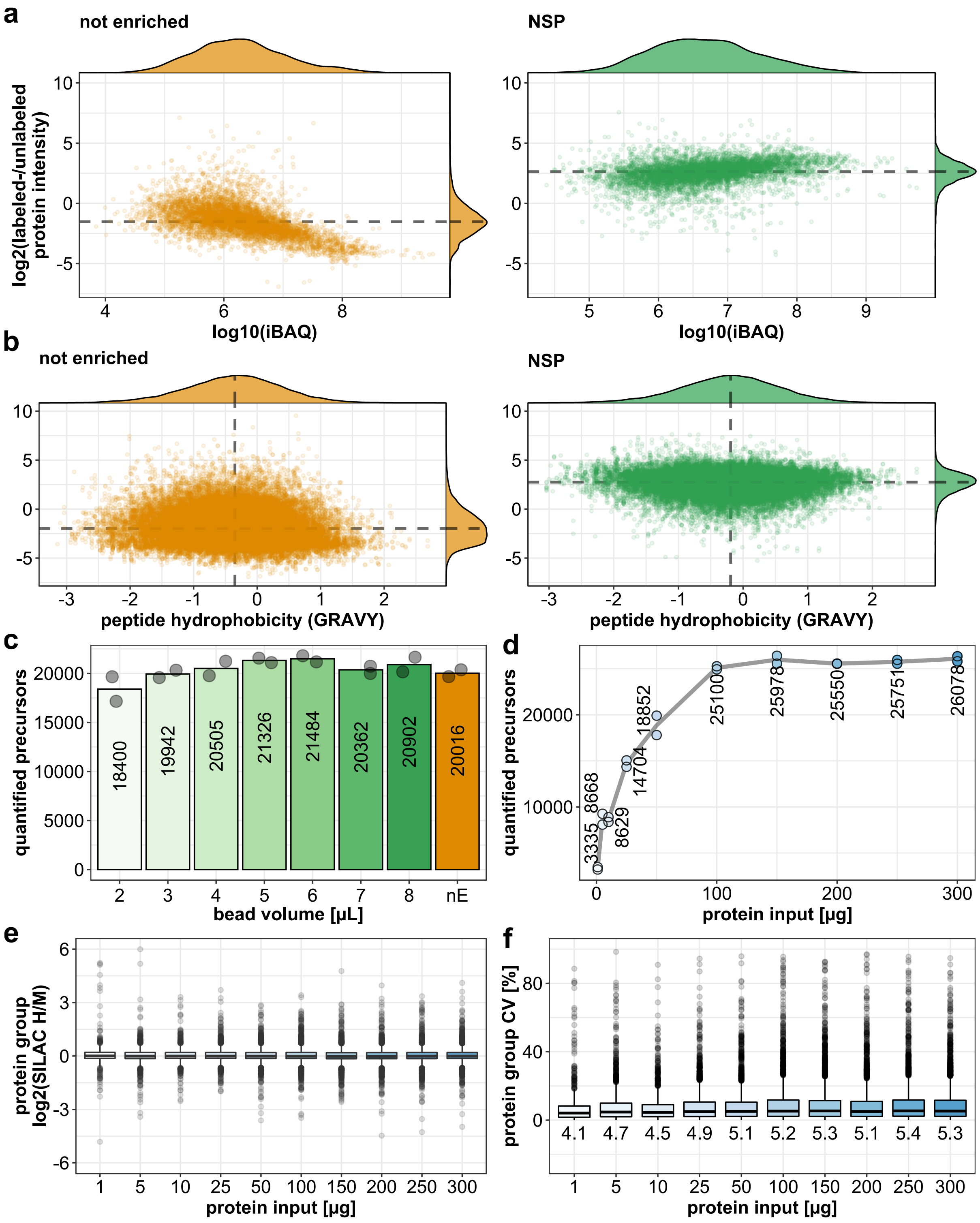

Supplementary Figure 2

Protein metrics of pulse-labeled samples analysed by mass spectrometry with and without prior enrichment of NSPs. A) Ratios of labelled NSPs over unlabelled pre-existing proteins, of enriched newly synthesized proteome samples and samples prepared without NSP enrichment plotted against iBAQ values of the respective protein group. B) Scatter plot of the precursor ratios of NSPs over pre-existing proteins, and the

respective peptide hydrophobicity (GRAVY index), in enriched newly synthesized proteome samples and samples prepared without NSP enrichment. Data show that NSPs span the full range in the GRAVY index. C) Number of quantified precursors (precursors with H/M SILAC ratio) in samples prepared with different amounts of MAA beads. D) Number of quantified precursors in samples prepared with different amounts of protein input. E) Boxplots of SILAC ratios of newly synthesized proteome samples generated with different amounts of protein input. H- and M-labelled samples were mixed in equal amounts, to produce an expected log2 H/M ratio of 0. Upper and lower whiskers of the boxplots extend from the hinges to the highest or lowest values that are within 1.5x the interquartile range. Values outside this range are plotted as dots and represent outliers. F) Boxplots indicating the coefficient of variation (CV) values calculated from the SILAC ratios of NSP samples that were prepared with different amounts of protein input. Upper and lower whiskers extend from the hinges to the highest or lowest values that are within 1.5x the interquartile range. Values outside this range are plotted as dots and represent outliers. Data are based on 2 experimental replicates.

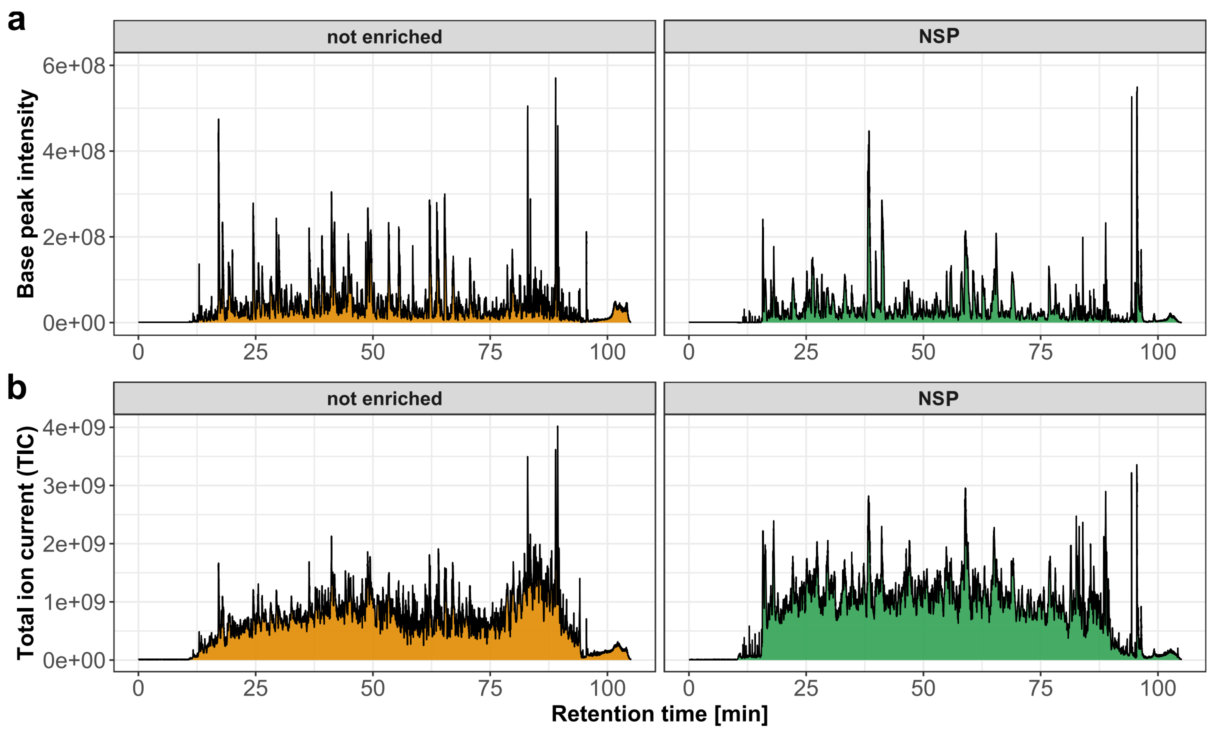

Supplementary Figure 3

Representative chromatograms of NSP samples, with (left panels) and without (right panels) enrichment of NSPs via click-chemistry. LC-MS analyses used identical LC gradients and mass spectrometer settings. A) Base peak- and B) Total ion current (TIC) chromatogram.

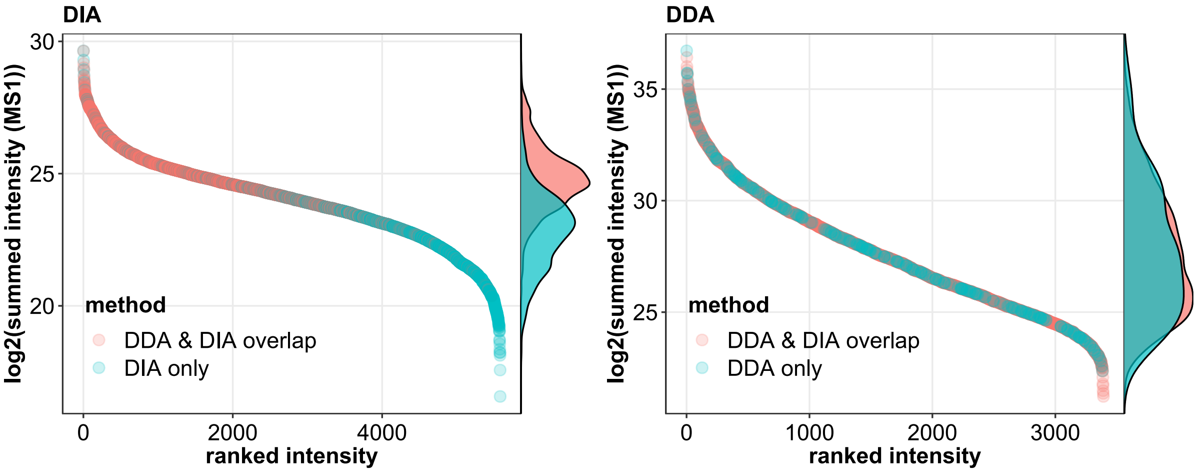

Supplementary Figure 4

Comparison of the summed MS1 intensities in the DDA and DIA data of the SILAC benchmark data. Overlaps and unique protein identifications between the different methods and their intensity distributions are indicated.

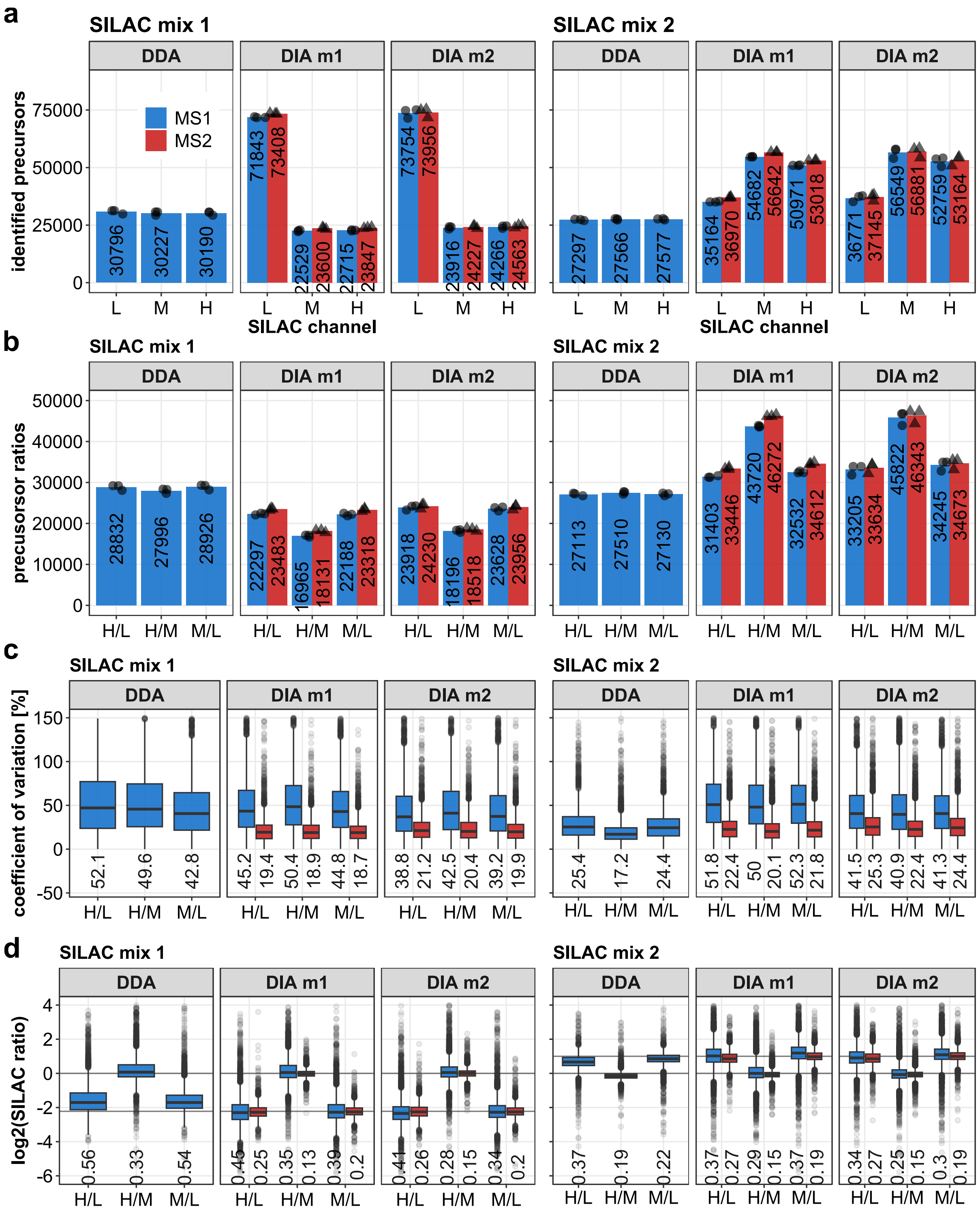

Supplementary Figure 5

Comparative analysis of SILAC labelled benchmark samples using data-dependent acquisition (DDA) and data-independent acquisition (DIA) mass spectrometry. A) Number of identified precursors in the light- (L), intermediate- (M) and heavy (H) SILAC channels. B) Precursor SILAC ratio numbers. Values based on MS1-based quantification in blue and MS2-based quantification indicated in red. C) Coefficient of variation (CV) values of the precursor SILAC ratios. D) Boxplots indicating the distribution of log2-transformed

precursor SILAC ratios. The median of the difference from the theoretical log2 ratio is indicated below the boxplots. Upper and lower whiskers extend from the hinges to the highest or lowest values that are within 1.5x the interquartile range. Values outside this range are plotted as dots and represent outliers. Data are based on 3 technical replicates of the single SILAC mix samples.

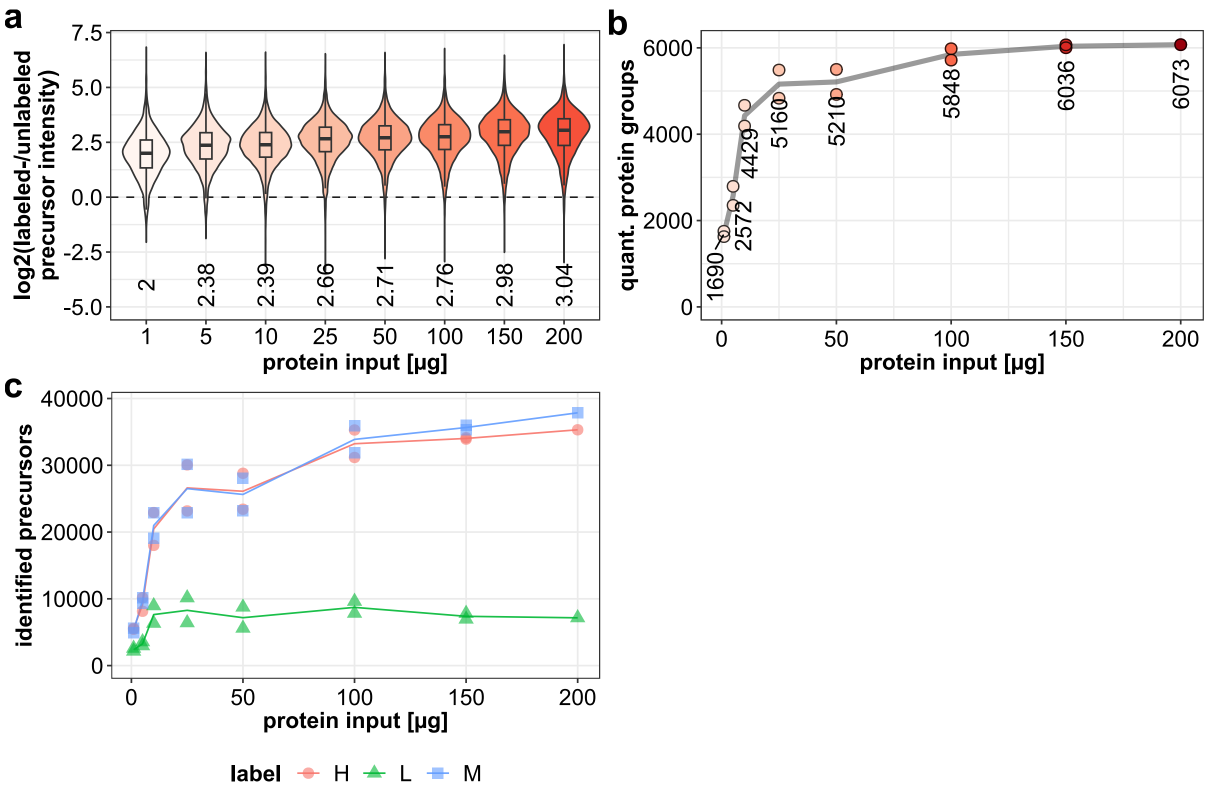

Supplementary Figure 6

Applying plexDIA for the analysis of samples prepared with the semi-automated NSP enrichment and assessing the ideal protein input range. A) Intensity ratios of samples prepared with differeing amounts of protein input. Heavy- and intermediate SILAC labelled precursors (originating from newly synthesized proteins), over light precursor ions (originating from pre-existing proteins). The upper and lower whiskers, of the ratio boxplots, extend from the hinges to the highest or lowest values that are within 1.5x the interquartile range. B) Number of quantified protein groups in the NSP samples prepared with differing protein input amonts. Numbers indicate the average of 2 replicates (red dots). C) Number of identified precursors with light- (L), intermediate- (M) and heavy (H) SILAC labels, in enriched samples with different amounts of protein input, analysed using plexDIA. Data are based on 2 technical replicates, except the 200 µg sample which consists of a single replicate.

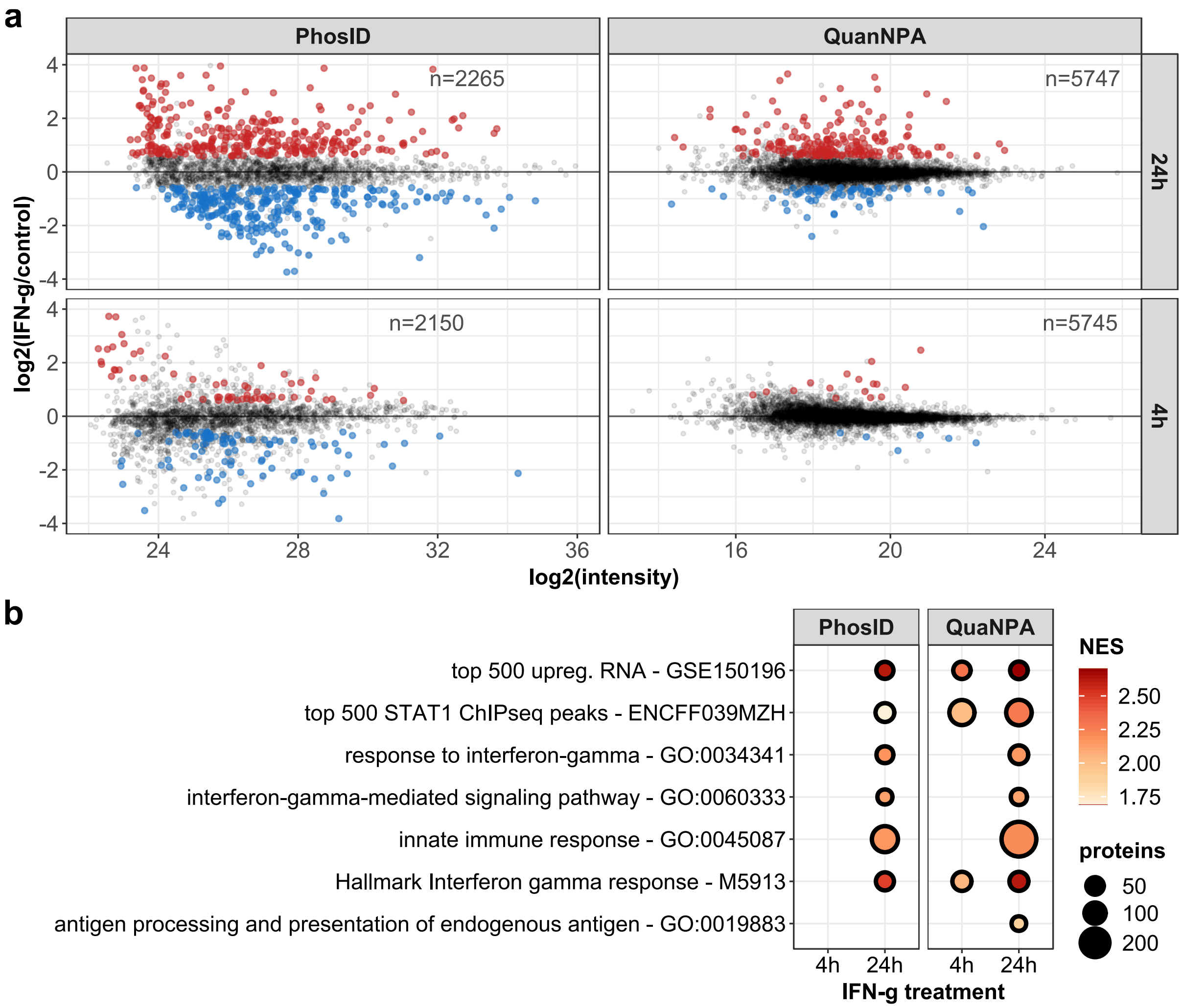

Supplementary Figure 7

Comparison of NSP data of IFNg-treated Hela cells, obtained with the QuaNPA workflow and with PhosID [24]. A) MA plots of newly synthesized proteome data of IFNg treated Hela cells, generated with the PhosID and QuaNPA workflow at two time points. The number of quantified proteins and differentially expressed proteins (adj. p-value < 0.05 & absolute log2FC > 0.585) are highlighted. Only unique protein groups from the samples generated with the QuaNPA workflow were included. PhosID samples were generated with 500 µg protein input and were measured using a 120 min method on an Orbitrap Fusion mass spectrometer (Thermo Fischer). QuaNPA samples were generated from 100 µg protein input and measured using a 90 min method on a QExactive HF mass spectrometer (Thermo Fischer). B) Dotplot representing GSEA results of the NSP data generated with the 2 methods. Only significantly enriched protein sets (q-value < 0.05) are included in the graph. Data are based on 3 experimental replicates.

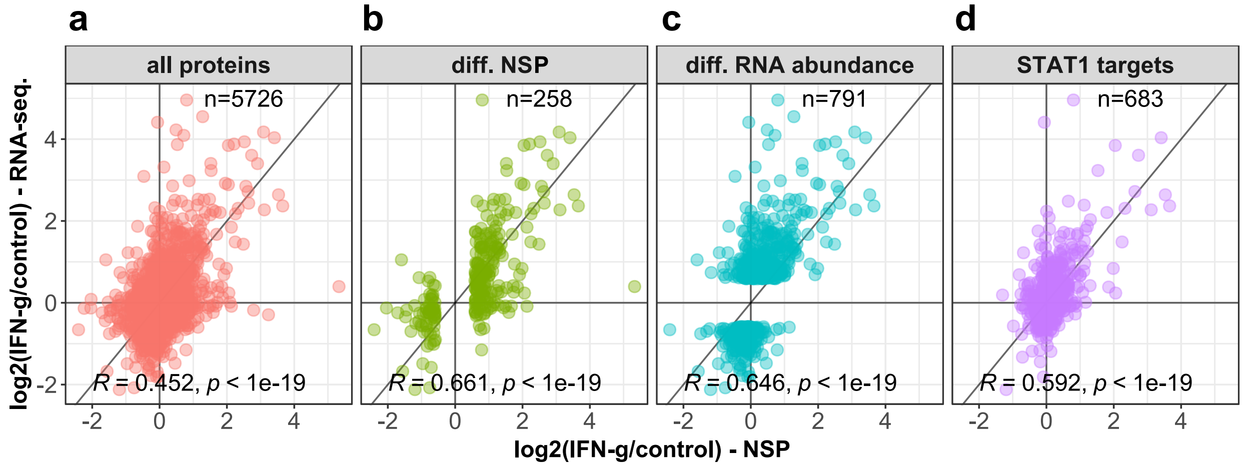

Supplementary Figure 8

Log2-transformed fold change values of the newly synthesized proteome (NSP) data and published RNAseq data of Hela cells treated with IFNg for 24 h [47], displayed in scatter plots. A) Correlation (Pearson) of the complete transcriptome and NSP data. B) Correlation (Pearson) of the subset of differentially expressed NSP subset (adj. p-value < 0.05 & abs(log2FC > 0.585)) and the respective transcriptomic data. C) Correlation (Pearson) of the subset of differentially expressed mRNA subset (adj. p-value < 0.05 & abs(log2FC > 0.585)) and the respective NSP data. D) Correlation (Pearson) of the subset of NSP and mRNA that are STAT1 ChIPseq targets in Hela cells treated with IFNg for 30 min.

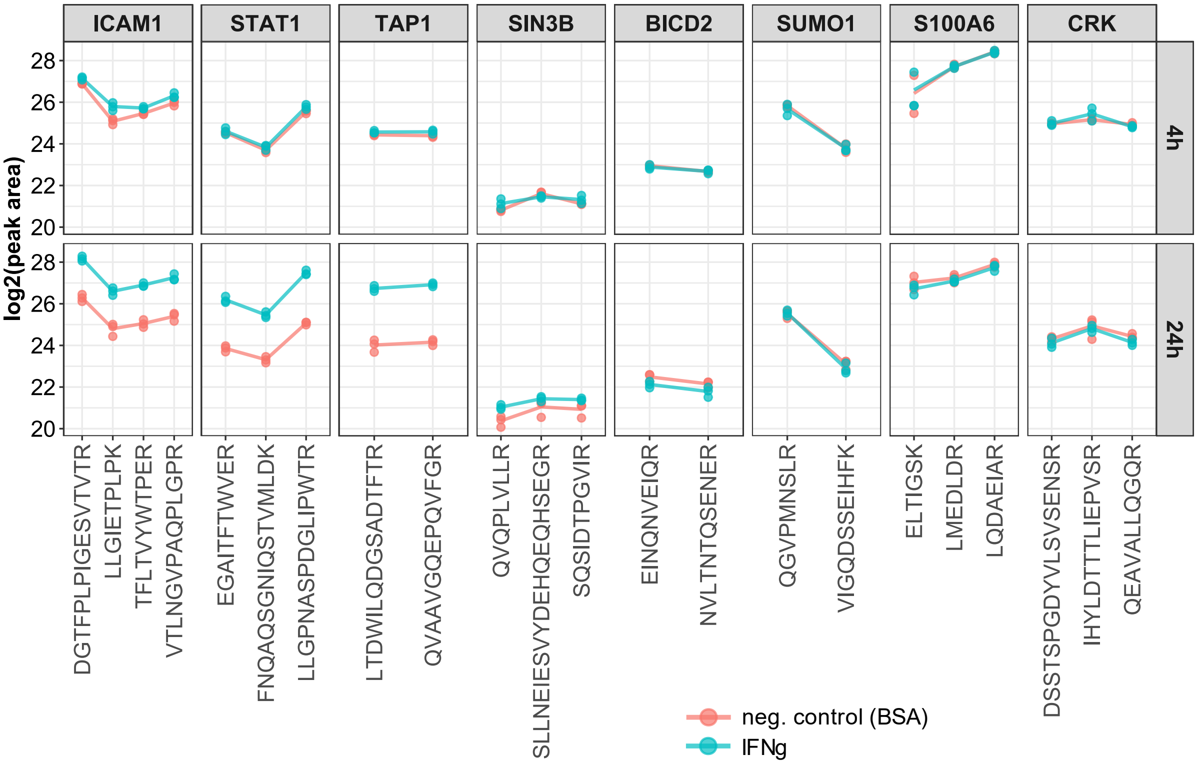

Supplementary Figure 9

Overview of the TIC normalized peak areas of the targeted peptides, analysed in Hela cells treated with IFNg or 0.5% BSA for 4 h and 24 h, using PRM measurements. Data are based on 3 experimental replicates.

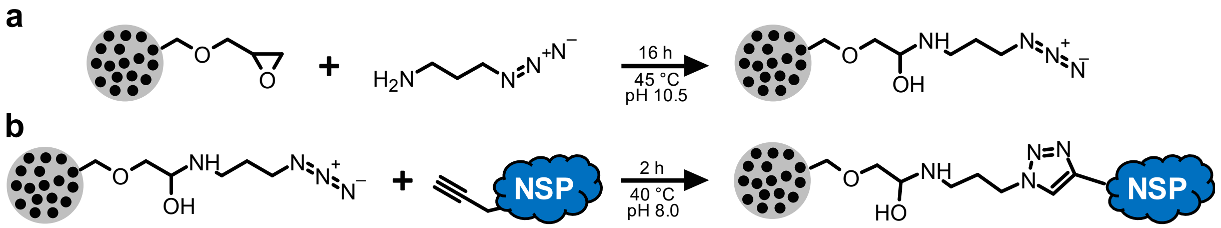

Supplementary Figure 10

Preparation and use of magnetic azide agarose (MAA) beads. A) Proposed coupling mechanism for Epoxy-activated magnetic agarose beads with 3-Azido-1-propanamine to produce MAA beads. B) Newly synthesized proteins, containing O-propargyl-puromycin (OPP), L-Homopropargylglycine (HPG) or β-Ethynylserine (β-ES), can be covalently bound to MAA beads using click chemistry (Cu(I)-catalyzed azide alkyne cycloaddition (CuAAC)).
