# Supplementary material for: An integrated workflow for quantitative analysis of the newly synthesized proteome
Source: Nat Commun. 2023 Dec 12;14:8237. doi: 10.1038/s41467-023-43919-3 (PMC10716174; doi:10.1038/s41467-023-43919-3)
Supplement: Supplementary file 3 — Description of Additional Supplementary Files [file 41467_2023_43919_MOESM3_ESM.pdf]

Description of Additional Supplementary Files:

Supplementary Data 1: Long formatted table containing the quantitative proteomics data from the initial optimizations of the semi-automated NSP enrichment protocol with different MAA bead volumes used for the enrichment (and contrast to a sample prepared without NSP enrichment).

Supplementary Data 2: Long formatted table containing the quantitative proteomics data from the initial optimizations of the semi-automated NSP enrichment protocol with different protein input amounts used for the enrichment.

Supplementary Data 3: Long formatted table containing the quantitative proteomics data from the benchmark of stable-isotope labelled Hela cell lysate mixtures, using different MS acquisition methods and analysis software for SILAC DDA and plexDIA analysis.

Supplementary Data 4: Wide formatted table containing the NSP log2 fold change values of all replicates of Hela cells, treated with IFNg at different time points.

Supplementary Data 5: Long formatted table containing the output tables of the differential expression analysis of the quantitative newly synthesized proteome data of IFNg-treated Hela cells, generated by DEqMS.

Supplementary Data 6: Table containing the log2 fold change values of top 500 upregulated genes (according to fold change) in IFNg-treated Hela cells, determined via RNA-sequencing and calculated by DESeq2. The data was retrieved and modified from NCBI database using accession number GSE150196, more specifically "GSE150196_RNA-seq_DESeq2_priming_vs_naive.tab".

Supplementary Data 7: Table containing the peak intensity values of top 500 gene promoters (according to signal value), bound by STAT1, in IFNg-treated Hela cells, determined via ChIP-seq. The data was retrieved and modified from the ENCODE database via accession number ENCSR000EZK, more specifically "ENCFF039MZH.bed".

Supplementary Data 8: Table containing the log2 fold change values and additional information on the subset of differentially regulated NSP, which have been highlighted in the heatmap in Figure 6.
