# Supplementary material for: An integrated workflow for quantitative analysis of the newly synthesized proteome
Source: Nat Commun. 2023 Dec 12;14:8237. doi: 10.1038/s41467-023-43919-3 (PMC10716174; doi:10.1038/s41467-023-43919-3)
Supplement: Supplementary file 12 — Reporting Summary [file 41467_2023_43919_MOESM12_ESM.pdf]

nature portfolio

Corresponding author(s): Jeroen Krijgsveld

Last updated by author(s): Oct 19, 2023

Reporting Summary

Nature Portfolio wishes to improve the reproducibility of the work that we publish. This form provides structure for consistency and transparency in reporting. For further information on Nature Portfolio policies, see our Editorial Policies and the Editorial Policy Checklist.

Statistics

For all statistical analyses, confirm that the following items are present in the figure legend, table legend, main text, or Methods section.

n/a	Confirmed	
☐	☒	The exact sample size (*n*) for each experimental group/condition, given as a discrete number and unit of measurement
☐	☒	A statement on whether measurements were taken from distinct samples or whether the same sample was measured repeatedly
☐	☒	The statistical test(s) used AND whether they are one- or two-sided<br>*Only common tests should be described solely by name; describe more complex techniques in the Methods section.*
☒	☐	A description of all covariates tested
☐	☒	A description of any assumptions or corrections, such as tests of normality and adjustment for multiple comparisons
☒	☐	A full description of the statistical parameters including central tendency (e.g. means) or other basic estimates (e.g. regression coefficient) AND variation (e.g. standard deviation) or associated estimates of uncertainty (e.g. confidence intervals)
☒	☐	For null hypothesis testing, the test statistic (e.g. *F*, *t*, *r*) with confidence intervals, effect sizes, degrees of freedom and *P* value noted<br>*Give P values as exact values whenever suitable.*
☒	☐	For Bayesian analysis, information on the choice of priors and Markov chain Monte Carlo settings
☒	☐	For hierarchical and complex designs, identification of the appropriate level for tests and full reporting of outcomes
☒	☐	Estimates of effect sizes (e.g. Cohen's *d*, Pearson's *r*), indicating how they were calculated

Our web collection on statistics for biologists contains articles on many of the points above.

Software and code

Policy information about availability of computer code

Data collection: Orbitrap Tune (2.11 QF2 Build 30007) and XCalibur (4.4) software from Thermo Fischer Scientific were used for data acquisition.

Data analysis: Raw MS data from DDA acquisition methods was processed using Maxquant (2.0.3), MS data from PRM acquisition was processed using Skyline (22.2.0.527) and Raw MS data from DIA acquisition methods was processed with DIA-NN (1.8.1). Further processing of the data was carried out using R (4.0.3) and custom scripts, which have been deposited via github (https://github.com/krijgsveld-lab/QuaNPA). LFQ normalization was carried out using the iq package (1.9.6). Differential expression analysis was performed using the limma (3.46.0) and DEqMS (1.8.0) R-packages. Overrepresentation enrichment analysis was carried out using a hypergeometric test via the "enricher" function of the clusterProfiler (3.18.0) R/Bioconductor package. Gene set enrichment analysis was carried out using the sorted log2 fold change values of all quantified protein groups, using the "GSEA" and "gseGO" function of the clusterProfiler (3.18.0) R/Bioconductor package. Gene lists of the Molecular Signatures Database were retrieved and analysed using the msigdbr (7.5.1) R package of the CRAN software repository. Peptide hydrophobicity (GRAVY index) was calculated using the "hydrophobicity" function of the Peptides (2.4.4) R package. Principal component analysis (PCA) of the log2 transformed SILAC ratios was performed using the "prcomp" function of the stats (4.0.3) R package. Differential protein abundance testing of the TIC-normalized PRM data, was carried out using MSstats (version 4.2.2.0).

For manuscripts utilizing custom algorithms or software that are central to the research but not yet described in published literature, software must be made available to editors and reviewers. We strongly encourage code deposition in a community repository (e.g. GitHub). See the Nature Portfolio guidelines for submitting code & software for further information.

Data

Policy information about availability of data

All manuscripts must include a data availability statement. This statement should provide the following information, where applicable:

- Accession codes, unique identifiers, or web links for publicly available datasets
- A description of any restrictions on data availability
- For clinical datasets or third party data, please ensure that the statement adheres to our policy

The mass spectrometry data, processing parameters and the respective SwissProt database used for the processing of the raw data viaMaxquant, DIA-NN and Skyline search engines have been deposited to the ProteomeXchange Consortium via the PRIDE and Panorama Public repository. The data from the initial method optimizations are available with identifier PXD036886, data from the SILAC labelled benchmark samples with identifier PXD039580 and PXD039578, and data from the IFNg time course experiment with identifier PXD038915. Raw files and data from the PRM analysis are available with ProteomeXchange identifier PXD043967. Raw files and data from the plexDIA input dilution analysis is available with PRIDE identifier PXD043817. RNA sequencing data, from Hela cells treated with IFNg, were retrieved from GSE150196. STAT1 ChIP-seq data, of Hela cells treated with IFNg, was retrieved from the ENCODE database via identifier ENCSR000EZK.

Research involving human participants, their data, or biological material

Policy information about studies with human participants or human data. See also policy information about sex, gender (identity/presentation), and sexual orientation and race, ethnicity and racism.

Reporting on sex and gender	N/A
Reporting on race, ethnicity, or other socially relevant groupings	N/A
Population characteristics	N/A
Recruitment	N/A
Ethics oversight	N/A

Note that full information on the approval of the study protocol must also be provided in the manuscript.

Field-specific reporting

Please select the one below that is the best fit for your research. If you are not sure, read the appropriate sections before making your selection.

[x] Life sciences   [ ] Behavioural & social sciences   [ ] Ecological, evolutionary & environmental sciences

For a reference copy of the document with all sections, see nature.com/documents/nr-reporting-summary-flat.pdf

Life sciences study design

All studies must disclose on these points even when the disclosure is negative.

Sample size	No statistical method was used to predetermine sample size. The number of experimental and technical replicates was based on considerations from previous experiments.
Data exclusions	Due to low confidence identifications in the PRM analysis, KLF3 precursors were excluded from downstream analysis, otherwise no data were excluded from the analysis in this study.
Replication	2 technical replicate samples were used to assess the quantitative reproducibility of the automated sample preparation protocol, 3 technical replicate measurements were used for the benchmarking of LC-MS methods and plexDIA analysis tools and 3 biological replicates were used for the final IFNg time course analysis. High quantitative reproducibility was observed for all analyzed data. Additionally, data from the IFN-gamma treated Hela cells was compared to reported data from multiple datasets, obtained with a diverse range of analytical methods.
Randomization	The 96-well plate positions and measurement order for the IFNg-treatment experiment were randomized. To avoid potential carry over effects in the LC-MS measurements, samples from the dilution series of magnetic alkyne agarose beads and protein input were acquired in ascending order.
Blinding	Since the samples were prepared and analyzed by the same researcher, blinding would have prohibited tracing of the produced data.

Reporting for specific materials, systems and methods

We require information from authors about some types of materials, experimental systems and methods used in many studies. Here, indicate whether each material, system or method listed is relevant to your study. If you are not sure if a list item applies to your research, read the appropriate section before selecting a response.

Materials & experimental systems

n/a	Involved in the study
☒	☐ Antibodies
☐	☒ Eukaryotic cell lines
☒	☐ Palaeontology and archaeology
☒	☐ Animals and other organisms
☒	☐ Clinical data
☒	☐ Dual use research of concern
☒	☐ Plants

Methods

n/a	Involved in the study
☒	☐ ChIP-seq
☒	☐ Flow cytometry
☒	☐ MRI-based neuroimaging

Eukaryotic cell lines

Policy information about cell lines and Sex and Gender in Research

Cell line source(s)	Hela cells (CCL-2) were obtained from ATCC.
Authentication	Cell line was not authenticated.
Mycoplasma contamination	Cell line was not tested for Mycoplasma contamination.
Commonly misidentified lines (See ICLAC register)	To the best of our knowledge no misidentified cell lines have been used in this study.
